# The effect of iron treatment on fetal fraction according to gestational weeks in maternal anemia

**DOI:** 10.3389/fmed.2026.1772334

**Published:** 2026-03-25

**Authors:** Seniye Burcu Torumtay Alic

**Affiliations:** Department of Gynecology and Obstetrics, Hitit University Faculty of Medicine, Çorum, Türkiye

**Keywords:** cell-free DNA, erythropoiesis, fetal fraction, gestational age, iron supplementation, maternal anemia, noninvasive prenatal testing

## Abstract

**Objective:**

The aim of this study was to evaluate the effects of iron (Fe) supplementation in maternal anemia on fetal fraction (FF) according to different gestational ages (GA) in pregnancies undergoing non-invasive prenatal testing (NIPT). FF is one of the key parameters that directly determines the analytical reliability of NIPT. Therefore, understanding the impact of hematologic and treatment-related variables on FF is important for optimal test timing and for minimizing false-negative results.

**Methods:**

This retrospective, single-center study included 308 pregnant women who underwent NIPT between January 2020 and December 2025. Cases with obesity, multiple pregnancies, *in vitro* fertilization conception, chronic systemic diseases, or medication use were excluded to obtain a homogeneous cohort. Hematologic parameters and FF values were evaluated according to GA and Fe supplementation status. Correlation, subgroup, and multivariable regression analyses were performed using non-parametric statistical methods.

**Results:**

The median maternal age was 33.5 years, and the median GA was 14.43 weeks. A moderate positive correlation was observed between GA and FF (*ρ* = 0.399, *p* < 0.001), whereas Hb levels showed only a weak positive correlation with FF (*ρ* = 0.112, *p* = 0.050). No significant associations were found between FF and hematocrit, white blood cell, or platelet counts. The median FF was significantly higher after 16 weeks of gestation (11.7% vs. 8.3%, *p* < 0.001). Notably, among pregnancies ≥16 weeks, FF was significantly lower in women receiving Fe supplementation compared with those who did not (*p* = 0.010), whereas no significant difference was observed before 16 weeks. In multivariable regression analysis adjusting for GA, Hb level at the time of NIPT, and MA, only GA remained independently associated with FF.

**Conclusion:**

Although crude analyses suggested lower FF values among women receiving Fe supplementation after 16 weeks of gestation, multivariable regression analysis indicated that GA remained the primary determinant of FF. Therefore, the apparent association between Fe supplementation and FF likely reflects underlying GA-related and hematologic differences rather than a direct effect of Fe therapy.

## Introduction

Today, non-invasive prenatal testing (NIPT) can detect not only common chromosomal abnormalities such as trisomy 21, 18, and 13, but also sex chromosome abnormalities, rare autosomal anomalies, and subchromosomal copy number variations (1–3).

Fetal fraction (FF) is defined as the proportion of fetal DNA within the total cfDNA present in maternal plasma, and it is one of the most critical parameters determining the sensitivity and specificity of NIPT. As the FF increases, the test’s ability to distinguish euploid from aneuploid pregnancies improves. Achieving an adequate amount of cfDNA is essential to prevent false-negative results and test failures (4).

Studies have shown that cfDNA fragments of fetal origin are shorter than maternal cfDNA fragments, and these shorter fragments offer an advantage in amplification and analysis (1, 2, 5). Although not yet applied in routine clinical practice, emerging enrichment techniques have been reported to potentially increase FF and reduce test failures (1, 3, 5). However, these methods remain limited by cost, processing time, and accessibility.

Therefore, identifying biological factors that influence FF continues to be clinically relevant. Predicting test failure in advance is valuable to prevent unnecessary anxiety, time loss, and financial burden for patients. Previous studies have demonstrated that factors such as gestational age (GA), maternal age (MA), history of *in vitro* fertilization (IVF), multiple pregnancy, chronic diseases, medication use, smoking, body mass index (BMI), lipid profile, and alanine aminotransferase (ALT) levels may affect FF (1, 6–9). Despite controlling for these factors, the NIPT failure rate remains around 1% (10), suggesting that additional biological parameters may play a role. Hemoglobinopathies such as thalassemia are relatively common in Mediterranean populations, including Türkiye, where carrier frequencies have been reported to range between approximately 2 and 5% (11). These conditions may influence maternal hematologic parameters and could potentially affect circulating cell-free DNA dynamics.

DNA methylation studies have shown that leukocytes of the hematopoietic system constitute a major component of the circulating cfDNA pool (12, 13). Qiao et al. (1) reported that FF decreases as maternal white blood cell (WBC) count increases. Although mature erythrocytes are anucleated, the erythroid lineage represents the largest cellular population within the hematopoietic system. During the transformation of erythroblasts into reticulocytes in the bone marrow, nuclear material is phagocytosed, and DNA fragments are released into the circulation (12, 14–17). It has been reported that approximately 30% of plasma DNA in healthy individuals is of erythroid origin (12). Based on these findings, we hypothesized that erythroid-derived maternal cfDNA may influence the FF.

In this study, we aimed to evaluate the effect of iron (Fe) therapy on FF before and after the onset of physiological hemodilution in pregnancies undergoing NIPT. We investigated whether Fe supplementation during pregnancy, by enhancing bone marrow erythropoiesis, could alter FF levels. Given the high prevalence of maternal anemia in developing countries, we additionally explored whether pretest complete blood count (CBC) parameters might serve as potential preanalytical indicators of FF variability.

## Materials and methods

This single-center, retrospective study included 308 pregnant women who underwent NIPT at our clinic between January 2020 and December 2025 and were found to be negative for chromosomal abnormalities. Only participants whose clinical records and laboratory data were fully accessible and who delivered at our institution were included. Because this was a retrospective study, no *a priori* sample size calculation was performed. A *post hoc* sensitivity analysis indicated that with a total sample size of 308 participants, the study had 80% statistical power to detect correlations of approximately |*ρ*| ≥ 0.16. For the ≥16-week subgroup comparison (*N* = 142), the study had sufficient power to detect small-to-moderate effect sizes (approximately *r* ≥ 0.24) in Mann–Whitney U tests.

Cases were excluded if they had a history of smoking, multiple pregnancy, IVF, chronic systemic diseases (such as diabetes mellitus, hypertension, or autoimmune disorders), or if they were using immunosuppressive agents, steroids, aspirin, or low-molecular-weight heparin. Patients with a BMI > 25, ultrasonographic or genetic evidence of fetal anomaly, incomplete data, or antenatal follow-up conducted elsewhere were also excluded.

For all participants, maternal CBC and NIPT data obtained on the same day were used for analysis. Recorded parameters included MA (years), GA (weeks + days), Fe supplementation status, and CBC values including hemoglobin (Hb, g/dL), hematocrit (Hct, %), WBC (×10^9^/L), and platelet count (PLT, ×10^9^/L), as well as NIPT-derived FF (FF, %).

CBC count analyses were performed using an automated hematology analyzer (Sysmex XN-1000, Sysmex Corporation, Kobe, Japan), which performs standardized measurements based on fluorescence flow cytometry technology. The laboratory reference ranges used in our institution were as follows: Hb 12–16 g/dL, Hct 36–46%, WBC 4–10 × 10^9^/L, and PLT 150–400 × 10^9^/L.

Participants were first evaluated based on their routine first-trimester CBC results. Those with an Hb level <11 g/dL were diagnosed with anemia and prescribed 100 mg of ferrous fumarate daily (equivalent to elemental divalent Fe), administered regularly for 3–4 weeks. On the day of NIPT sampling, CBC was repeated, and study parameters were recorded. Participants were classified into two main groups according to Fe therapy status (Fe users vs. non-users) and into subgroups based on GA (<16 weeks vs. ≥16 weeks).

The 16th gestational week corresponds to the period when physiological hemodilution begins and erythropoiesis in the bone marrow increases (13, 18, 19); therefore, it was used as the landmark point in this study.

All NIPT analyses were performed by the same reference laboratory using the NIPT ONE PLUS test (Nevagen Genetik, Istanbul, Türkiye). Cell-free DNA was extracted from maternal plasma collected in Streck tubes and transported under manufacturer-recommended conditions. Sequencing was performed using a massively parallel sequencing platform based on DNBSEQ™ technology (MGI DNBSEQ-T7RS, MGI Tech Co., Shenzhen, China). FF was estimated using the platform’s validated bioinformatic algorithm according to the laboratory’s analytical workflow.

Maternal Hb levels were also categorized into clinically relevant intervals (8–9, 9–10, 10–11, 11–12, 12–13, 13–14, and >14 g/dL) to visualize the distribution of FF across Hb categories.

Among women with baseline anemia (Hb < 11 g/dL), an additional subgroup analysis was conducted to evaluate the effect of Hb correction following Fe supplementation. Participants were categorized as having persistent anemia (Hb < 11 g/dL at NIPT) or corrected anemia (Hb ≥ 11 g/dL at NIPT).

### Statistical analysis

All statistical analyses were performed using IBM SPSS Statistics for Windows, Version 22 (IBM Corp., Armonk, NY, USA). Data distribution was assessed with the Kolmogorov–Smirnov test and visual inspection of histograms. Since the data were not normally distributed, non-parametric tests were applied. Descriptive statistics were presented as median and interquartile range (IQR) for continuous variables, and as frequency (percentage) for categorical variables. Spearman’s rank correlation coefficient (*ρ*) was used to evaluate correlations between FF and maternal clinical or hematological parameters, including MA, GA, Hb, Hct, WBC count, and platelet count. Comparisons between two independent groups were performed using the Mann–Whitney U test, and effect sizes were calculated using the formula *r* = *Z*/√*N*. FF values were also compared according to maternal Hb status at the time of NIPT (Hb < 11 g/dL vs. Hb ≥ 11 g/dL). Among women with baseline anemia, FF values were additionally compared between those with persistent anemia and those with corrected Hb levels after Fe supplementation. Multivariable linear regression analyses were conducted to identify independent predictors of FF. Because baseline Hb and Hb measured at the time of NIPT were highly correlated, they were evaluated in separate regression models to avoid multicollinearity. Model 1 included GA, baseline Hb, MA, and Fe supplementation. Model 2 included GA, Hb measured at the time of NIPT, MA, and Fe supplementation. Regression coefficients (B), standardized coefficients (*β*), and 95% confidence intervals (CI) were reported.

Multicollinearity among independent variables was assessed using variance inflation factors (VIF). Bootstrap resampling with 1,000 samples was used to estimate confidence intervals for regression coefficients. Boxplot visualizations showing the distribution of FF according to maternal Hb categories at baseline and at the time of NIPT were generated using R software (version 4.5.2; R Foundation for Statistical Computing, Vienna, Austria). All statistical tests were two-tailed, and a *p* value <0.05 was considered statistically significant.

## Results

A total of 308 pregnant women were included in the study, with a mean MA of 33.5 years and a median GA of 14.43 weeks. Among the participants, 166 (53.9%) were <16 weeks of gestation, and 142 (46.1%) were ≥16 weeks. The median FF value was 9.70% (IQR: 5).

Of all participants, 143 (46.4%) reported using Fe supplementation, whereas 165 (53.6%) did not. The median duration of Fe use was 23 days (IQR = 3) in both groups. The median Hb level was 11.70 g/dL (IQR: 2.70), Hct 35.15% (IQR: 7.28), WBC count 8.72 × 10^3^/μL (IQR: 3.14), and PLT count 242 × 10^3^/μL (IQR: 79.5) (Table 1).

**Table 1:** Descriptive characteristics of the study population (*n* = 308).

Variable	Category	Value
MA (years), median (IQR)		33.5 (8)
GA (weeks), median (IQR)		14.43 (5.97)
GA *f* (%)	<16 week	166 (53.9%)
	>16 week	142 (46.1%)
FF (%), median (IQR)		9.70 (5)
Fe supplementation *f* (%)	Yes	143 (46.4%)
	No	165 (53.6%)
Duration of Fe use (days), median (IQR)		23(3)
Baseline Hb (g/dL), median (IQR)		11.70 (2.70)
Hb at NIPT (g/dL), median (IQR)		11.75 (3.78)
Hct (%), median (IQR)		35.15 (7.28)
WBC count (×10^3^/μL), median (IQR)		8.72 (3.14)
PLT count (×10^3^/μL), median (IQR)		242 (79.5)

MA, Maternal Age; GA, Gestational Age; FF, Fetal Fraction; Fe, Iron; Hb, Hemoglobin; Hct, Hematocrit; WBC, White Blood Cell; PLT, Platelet count.

According to the Spearman correlation analysis, a moderate positive correlation was observed between GA and FF (*ρ* = 0.399, *p* < 0.001, 95% CI: 0.292–0.490). Baseline Hb showed a weak but statistically significant positive correlation with FF (*ρ* = 0.190, *p* = 0.001, 95% CI: 0.077–0.292). Hb measured at the time of NIPT demonstrated a weaker and borderline association with FF (*ρ* = 0.112, *p* = 0.050, 95% CI: −0.002–0.215). In contrast, no statistically significant correlations were identified between MA, Hct, WBC, or PLT and FF (Table 2).

**Table 2:** Spearman correlations between fetal fraction and maternal/hematologic variables.

Variable	FF (*ρ*, *p*)	95% CI
MA (years)	−0.096 (0.093)	−0.209 – 0.018
GA	0.399 (<0.001)	0.292–0.490
Baseline Hb	0.190 (0.001)	0.077–0.292
Hb at NIPT	0.112 (0.050)	−0.002 – 0.215
Hct	0.077 (0.177)	−0.031 – 0.187
WBC	0.029 (0.609)	−0.086 – 0.135
PLT	−0.083 (0.147)	−0.197 – 0.027

FF, Fetal Fraction; MA, Maternal Age; Hb, Hemoglobin; GA; Gestational Age; WBC, White Blood Cell; PLT, Platelet; NIPT, Non-invasive prenatal testing.

According to the Mann–Whitney U test results, the median FF value was significantly higher in pregnancies with a GA of ≥16 weeks compared to those <16 weeks (*U* = 5,722, *p* < 0.001).

Additionally, FF values were slightly higher in participants who did not receive Fe supplementation compared to those who did (*U* = 10,208, *p* = 0.041). There was no statistically significant difference in the duration of Fe supplementation between pregnancies before and after 16 weeks (*U* = 2301.5, *p* = 0.314) (Table 3).

**Table 3:** Fetal fraction by gestational age and iron supplementation.

Variable	Group	*N*	Median (IQR)	*U*	*p*
GA	<16 weeks	166	8.33 (3.99)	5,722	<0.001
	≥16 weeks	142	11.70 (5.35)		
Fe supplementation	No	165	10.04 (5.49)	10,208	0.041
	Yes	143	9.24 (5.01)		
Duration of Fe use (days)	<16 weeks	76	23 (3)	2301.5	0.314
	≥16 weeks	67	23 (3)		

GA, Gestational Age; Fe, Iron; The Mann–Whitney U test was used.

Baseline Hb levels differed markedly between the Fe-supplemented and non-supplemented groups. Median baseline Hb was 9.1 g/dL in the Fe-supplemented group and 13.0 g/dL in the non-supplemented group (*p* < 0.001). Hb levels remained significantly lower in the Fe-supplemented group at the time of NIPT sampling [10.1 g/dL (IQR 0.80)] compared with the non-supplemented group [12.8 g/dL (IQR 1.10)] (Table 4). These findings confirm that women receiving Fe supplementation represented a subgroup with substantially lower baseline Hb levels. The magnitude of these differences was large, with very strong effect sizes observed for both baseline and NIPT-day Hb levels (*r* = 0.86 for both comparisons).

**Table 4:** Baseline and NIPT-day hemoglobin levels according to iron supplementation.

Hemoglobin parameter	Fe (+) *n* = 143	Fe (−) *n* = 165	*U*	*Z*	*r*	*p*
Baseline Hb (g/dL), median (IQR)	9.1 (1.10)	13.0 (1.05)	0	−15.142	0.86	<0.001
Hb at NIPT (g/dL), median (IQR)	10.1 (0.80)	12.8 (1.10)	54	−15.071	0.86	<0.001

Hb, Hemoglobin; Fe, Iron supplementation; NIPT, Non-invasive prenatal testing. The Mann–Whitney U test was used.

In the subgroup analysis based on GA and Fe supplementation status, no statistically significant difference in FF was observed between supplemented and non-supplemented participants in pregnancies <16 weeks (*U* = 3250.50, *p* = 0.583). However, among pregnancies ≥16 weeks, FF was found to be significantly lower in the Fe-supplemented group (*U* = 1885.50, *p* = 0.010), with a small-to-moderate effect size (*r* = 0.21). When comparing GA across Fe supplementation status, FF increased significantly with advancing GA in both the supplemented (*U* = 1551.00, *p* < 0.001) and non-supplemented (*U* = 1244.00, *p* < 0.001) groups (Table 5).

**Table 5:** Fetal fraction comparison according to gestational age and iron supplementation.

GA	Fe supplementation	*N*	Median (IQR)	*U*	*Z*	*r*	*p*
<16 weeks	Yes	76	8.13 (4.02)	3250.50	−0.549	0.04	0.583
	No	90	8.35 (3.70)				
≥16 weeks	Yes	67	10.50 (5.81)	1885.50	−2.562	0.21	0.010
	No	75	12.57 (4.41)				
<16 weeks	Yes	76	8.13 (4.02)	1,551	−4.025		<0.001
≥16 weeks		67	10.50 (5.81)				
<16 weeks	No	90	8.35 (3.70)	1,244	−6.974		<0.001
≥16 weeks		75	12.57 (4.41)				

GA, Gestational Age; Fe, Iron supplementation. The Mann–Whitney U test was used.

A multivariable linear regression analysis was conducted to evaluate factors associated with FF. Because baseline Hb and Hb measured at the time of NIPT were highly correlated, they were evaluated in separate regression models to avoid multicollinearity.

In Model 1, which included GA, baseline Hb, MA, and Fe supplementation, the overall model was statistically significant (*R*^2^ = 0.186, adjusted *R*^2^ = 0.175, *p* < 0.001). GA was the only independent predictor of FF (*B* = 0.004, *β* = 0.375, 95% CI: 0.003–0.005, *p* < 0.001). Baseline Hb (*p* = 0.203), MA (*p* = 0.243), and Fe supplementation (*p* = 0.644) were not significantly associated with FF.

In Model 2, which included Hb measured at the time of NIPT instead of baseline Hb, similar results were observed. GA remained significantly associated with FF (*B* = 0.004, *β* = 0.396, 95% CI: 0.003–0.005, *p* < 0.001), whereas Hb level at NIPT (*p* = 0.532), MA (*p* = 0.201), and Fe supplementation (*p* = 0.140) were not independently associated with FF. The overall model explained approximately 18.3% of the variance in FF (*R*^2^ = 0.183; adjusted *R*^2^ = 0.173).

Variance inflation factors indicated no evidence of problematic multicollinearity in the final models (see Table 6).

**Table 6:** Multivariable linear regression analysis of factors associated with fetal fraction.

Variable	*B*	*β*	95% CI	*p*	VIF
Gestational age	0.004	0.375	0.003–0.005	<0.001	1.09
Baseline Hb	0.004	0.188	−0.002 – 0.009	0.203	8.04
Maternal age	0.000	−0.062	−0.001 – 0.000	0.243	1.03
Iron supplementation	0.005	0.067	−0.017 – 0.028	0.644	7.91

CI, confidence interval; Hb, hemoglobin; VIF, variance inflation factor. Bootstrap confidence intervals were calculated using 1,000 resamples.

Model 1, FF = 0.012 + (0.004 × GA) + (0.004 × Baseline Hb) + (0.000 × MA) + (0.005 × Fe Supplementation).

Model 2, FF = 0.080 + (0.004 × GA) + (−0.002 × HbNIPT) + (0.000 × MA) + (−0.013 × Fe Supplementation).

When participants were stratified according to Hb status at the time of NIPT, the median FF was 9.39% (IQR: 5.0) in the persistent anemia group (Hb < 11 g/dL) and 9.88% (IQR: 5.0) in those with Hb ≥ 11 g/dL, with no statistically significant difference observed (*U* = 12,548, *p* = 0.217).

Among women with baseline anemia (Hb < 11 g/dL), Hb levels improved to ≥11 g/dL in 12 participants after Fe supplementation, whereas anemia persisted in 131 participants. In this subgroup, the median FF was 9.39% (IQR: 5.0) in the persistent anemia group and 7.75% (IQR: 5.0) in the Hb-corrected group; however, this difference did not reach statistical significance (*U* = 549.5, *p* = 0.085) (Table 7).

**Table 7:** Fetal fraction according to hemoglobin status at NIPT and hemoglobin correction among women with baseline anemia.

Analysis	Hb status	*n*	Median FF (%) (IQR)	*U*	*p*
All participants (NIPT Hb status)	Persistent anemia (Hb < 11 g/dL)	131	9.39 (5.0)	12.548	0.217
	Hb ≥ 11 g/dL	177	9.88 (5.0)		
Baseline anemia subgroup	Persistent anemia (Hb < 11 → <11)	131	9.39 (5.0)	549.5	0.085
	Hb corrected (Hb < 11 → ≥11)	12	7.75 (5.0)		

**Figure 1:**
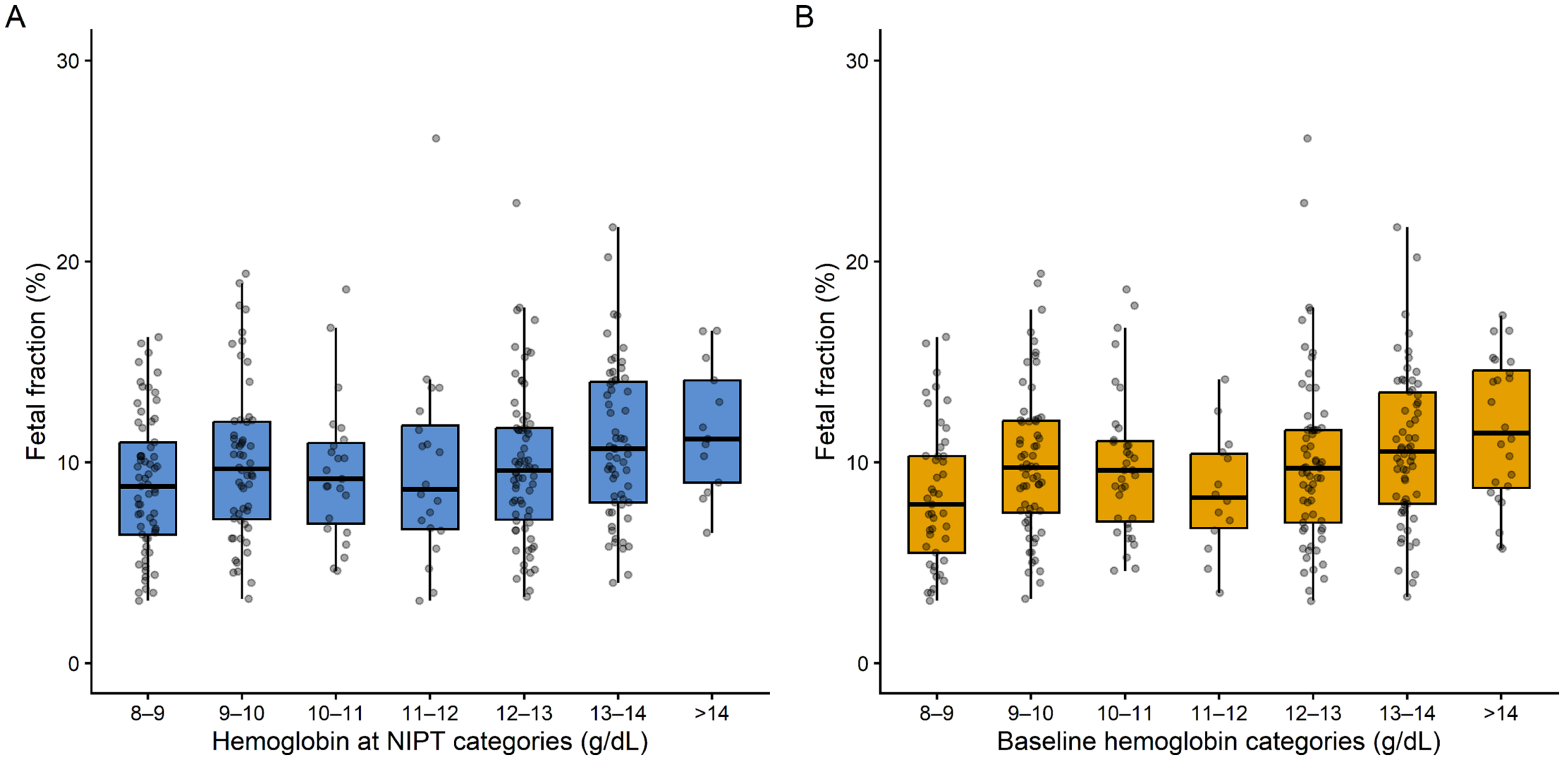
Distribution of fetal fraction according to maternal hemoglobin categories. (A) Hemoglobin categories at the time of NIPT. (B) Baseline hemoglobin categories. Boxplots represent the median and interquartile range, and individual points indicate participant-level observations.

The distribution of FF across maternal Hb categories measured at baseline and at the time of NIPT is illustrated in Figures 1A,B.

## Discussion

The present study investigated the potential influence of maternal hematologic parameters and Fe supplementation on FF in pregnancies undergoing NIPT, with particular attention to the modifying role of GA. Our findings demonstrated a significant positive correlation between GA and FF, whereas maternal Hb levels showed only a weak positive association with FF. In contrast, no significant relationships were observed between FF and MA, Hct, WBC, or PLT levels. Furthermore, FF values were significantly lower among women receiving Fe supplementation at or beyond the 16th gestational week. Taken together, these findings suggest that maternal hematologic parameters may act as potential preanalytical factors influencing FF; however, their independent contribution appears limited when GA is taken into account.

NIPT represents a groundbreaking screening method capable of detecting fetuses with chromosomal abnormalities at an early stage. FF refers to the proportion of fetal-derived cfDNA within the total cfDNA in maternal plasma, and the reliability of NIPT increases proportionally with higher FF levels. Numerous factors influencing FF have been identified. Studies have investigated various biological parameters such as MA, GA, BMI, smoking, multiple pregnancy, IVF conception, maternal systemic diseases, medication use, triglyceride and ALT levels, as well as WBC and PLT counts, to understand how they affect FF (1, 6–9, 14–16).

### Relationship between MA and FF

In a large cohort study conducted by Hou et al. (17), patients were divided into five age groups, and it was observed that FF decreased progressively with increasing MA. Although some studies have reported a significant effect of MA on FF, most investigations have found no meaningful correlation (14). In our study, MA did not show a significant impact on FF, which is consistent with the findings reported by Scott et al. (20).

### Relationship between GA and FF

In our study, consistent with the existing literature, FF levels were found to be higher in pregnancies at or beyond 16 weeks of gestation. As GA advances, the increase in placental volume and trophoblastic activity leads to greater amounts of fetal cfDNA entering the maternal circulation (2, 4, 15–17, 20). Although physiological hemodilution during pregnancy leads to progressive expansion of maternal plasma volume beginning in the late first trimester and becoming more pronounced in the second trimester (19, 21), FF continues to increase with advancing GA (15, 17). This apparent paradox may be explained by the concurrent increase in placental mass and trophoblastic turnover. As gestation progresses, enhanced trophoblast apoptosis contributes to greater release of fetal cell-free DNA into the maternal circulation (4). Consequently, the increasing contribution of fetal cfDNA appears to outweigh the dilutional effect of plasma volume expansion.

Wang et al. (16) reported that FF rises linearly between 10 and 22 weeks of gestation, with a more pronounced increase after the 15th week. Similarly, Hui and Bianchi (4) emphasized that the low fetal cfDNA levels in early pregnancy contribute to higher NIPT failure rates, suggesting that performing the test closer to the second trimester improves analytical reliability. Early gestational sampling has been identified as an important determinant of low FF and NIPT test failure (6, 10). Based on these findings, GA appears to be an important determinant of FF. However, whether performing NIPT after the 16th gestational week reduces the risk of low FF–related test failure requires confirmation in larger prospective studies.

### Clinical relevance of low fetal fraction and NIPT test failure

Low FF is recognized as one of the most important causes of NIPT failure or “no-call” results. Many NIPT platforms use a minimum FF threshold of approximately 2.8–4%, below which test accuracy decreases and the likelihood of test failure increases (4, 10). In the present cohort, although 11 cases (3.57%) had FF values below 4% (six in the Fe-supplemented group and five in the non-supplemented group), all tests were successfully reported and none required repeat sampling. Therefore, no actual NIPT test failures occurred in our cohort, and an analysis of failure rates was not possible. Nevertheless, the presence of cases with low FF values highlights the potential clinical relevance of factors influencing FF. In this context, the observed associations between maternal hematological parameters and FF may have implications for NIPT reliability and test performance in clinical practice, as low FF is a well-recognized contributor to reduced test performance and potential test failure (16).

### Effect of maternal hematological parameters on FF

The relationship between maternal hematological indicators and FF has been increasingly investigated in the literature. Qiao et al. (1) reported that elevated WBC counts (>12,000/μL) increase the amount of maternal DNA and thereby reduce FF. Similarly, Zhang et al. (22) demonstrated that high platelet distribution width values may adversely affect FF detection. In our study, no significant correlation was found between WBC or PLT and FF. However, since this study included a cohort with WBC values below 12,000/μL to specifically examine the effect of the erythroid series, the absence of a relationship with leukocytic variables was an expected finding.

In this study, a weak positive correlation was observed between Hb level and FF. The weak association between Hb level and FF reflects the multifactorial nature of FF (6–9). However, the increase in FF rate with rising Hb levels may be explained by a relative reduction in maternal cfDNA during the physiological compensation of anemia or by an increased proportion of DNA originating from the fetoplacental circulation (1, 12, 13, 21). The weak positive correlation between Hb level and FF reflects the complex effects of anemia on hematopoietic and cfDNA dynamics, and we believe that further prospective studies with serial measurements are needed to clarify the underlying mechanisms of this relationship. This pattern is also visually reflected in Figures 1A,B, where FF distributions across maternal Hb categories show a gradual upward tendency but with considerable overlap between groups, supporting the finding that Hb level is not an independent determinant of FF.

### Effect of Fe supplementation on FF according to GA

The erythroid lineage represents the largest cellular population in the bone marrow. Although circulating mature erythrocytes are anucleate, DNA methylation studies have demonstrated that approximately 30% of circulating maternal cell-free DNA originates from the erythroid lineage (12, 13). DNA fragments released during the differentiation of erythroblasts into reticulocytes and mature erythrocytes in the bone marrow contribute substantially to maternal cell-free DNA. Consequently, factors that increase bone marrow turnover could theoretically increase maternal cfDNA levels and potentially influence FF (12, 13, 23–25).

In the present study, the 16th gestational week was selected as a clinically meaningful threshold. Although physiological hemodilution begins in the first trimester, the most pronounced plasma volume expansion occurs during the early second trimester. Previous systematic reviews have demonstrated a marked increase in plasma volume between 14 and 20 weeks of gestation (18, 19, 21). In parallel, erythropoietic activity also tends to increase during this period as maternal hematologic adaptation progresses. Around the 16th gestational week, the combined effects of plasma volume expansion and enhanced erythropoietic stimulation may influence circulating maternal cfDNA dynamics. Based on the available literature, we interpret this gestational window as a potentially relevant period for evaluating the relationship between anemia, Fe supplementation, and FF.

Consistent with this physiological framework, our findings demonstrated no significant difference in FF between women with and without Fe supplementation before 16 weeks of gestation. However, among pregnancies at or beyond 16 weeks, FF was significantly lower in the Fe-supplemented group. This observation may be related to physiological hematological adaptations occurring during mid-pregnancy; however, the specific biological mechanisms remain uncertain.

Importantly, baseline hematologic characteristics differed substantially between the study groups. As shown in Table 4, women receiving Fe supplementation had markedly lower baseline Hb levels compared with those who did not receive supplementation. Although Hb levels increased following treatment, they remained significantly lower at the time of NIPT sampling in the supplemented group. These findings suggest that the Fe-treated cohort represents a biologically distinct subgroup characterized by baseline anemia. Therefore, the observed association between Fe supplementation and FF should be interpreted cautiously, as part of this relationship may reflect underlying hematologic differences rather than a direct pharmacologic effect of Fe supplementation. This interpretation is further supported by the multivariable regression analysis, which showed that Fe supplementation was not independently associated with FF after adjustment for GA and Hb levels.

Taken together, the combined results of correlation analyses, subgroup comparisons, graphical visualization, and multivariable regression consistently indicate that GA represents the primary biological determinant of FF.

To further clarify whether Fe supplementation independently influences FF, two multivariable regression models were constructed. Because baseline Hb and Hb measured at the time of NIPT were highly correlated, these variables were evaluated in separate models to avoid multicollinearity. In both models, GA remained the only variable independently associated with FF, whereas Hb levels and Fe supplementation were not significant predictors. These findings indicate that the apparent associations observed in unadjusted subgroup analyses likely reflect differences in GA distribution and baseline hematologic characteristics rather than a direct biological effect of Fe supplementation.

In addition, stratified analyses based on Hb status at the time of NIPT and Hb correction among women with baseline anemia did not demonstrate a significant association with FF. Participants with persistent anemia and those whose Hb levels increased to ≥11 g/dL showed comparable FF values. Similarly, among women with baseline anemia, correction of Hb following Fe supplementation was not associated with a measurable change in FF. These findings suggest that short-term hematologic improvement during pregnancy may not substantially alter circulating fetal cfDNA proportions, further supporting the notion that GA rather than dynamic maternal hematologic changes represents the dominant determinant of FF.

### Strengths and limitations of the study, future directions

The main strength of this study lies in investigating the relationship between FF and hematological variables within a homogeneous pregnancy population, excluding potential confounding factors such as maternal systemic diseases, obesity, and medication use. Moreover, to the best of our knowledge, no previous study in the literature has specifically examined the effect of Fe supplementation on FF according to GA.

However, this study also has several limitations. First, it was conducted as a single-center, retrospective study with a relatively limited sample size. Because the cohort represents a specific regional population, the regression model derived from these data should be interpreted cautiously in terms of generalizability. Given the demographic and regional variability across the seven geographical regions of Türkiye, multicenter studies including more diverse populations are needed to confirm these findings and establish broadly applicable predictive models.

Second, maternal ferritin levels and comprehensive Fe parameters could not be evaluated, and erythropoietic markers such as the reticulocyte count were not available. These biochemical parameters may provide additional insight into maternal hematologic status and its potential influence on circulating cell-free DNA dynamics.

In addition, several potential factors that could influence maternal hematological parameters and circulating cell-free DNA dynamics were not available in our retrospective dataset. Thalassemia carrier status, which is relatively prevalent in Mediterranean populations including Türkiye, may affect maternal hematologic indices and potentially influence circulating cfDNA proportions. However, systematic screening data for thalassemia were not available and therefore this variable could not be evaluated in the present analysis. Similarly, information regarding maternal–fetal blood group compatibility and Rh status was not consistently recorded and could not be incorporated into the statistical models.

Another contextual limitation relates to the accessibility of NIPT testing. In Türkiye, as in many middle-income countries, NIPT is primarily available through private healthcare services and is not universally reimbursed by the public healthcare system. Consequently, socioeconomic factors may influence access to testing, which could introduce a degree of selection bias in the studied cohort.

Finally, placental structural or functional parameters were not available in this dataset. Placental mass, trophoblastic turnover, and possible erythropoietic activity within placental tissues have been suggested as contributors to circulating fetal cfDNA release during pregnancy. Future prospective studies incorporating placental measurements and detailed hematological profiling may help clarify the biological mechanisms underlying variations in FF.

## Conclusion

In conclusion, this study suggests that GA is one of the most important determinants of FF in non-invasive prenatal testing. Although crude analyses suggested lower FF values among women receiving Fe supplementation, multivariable regression analysis showed that this association was not independent after adjustment for GA and Hb levels. Furthermore, Hb correction among women with baseline anemia was not associated with significant changes in FF. These findings suggest that maternal Fe supplementation and short-term hematologic improvement do not independently influence FF, and that GA remains the dominant biological factor affecting circulating fetal cell-free DNA levels in maternal plasma. Future prospective studies incorporating detailed Fe metabolism markers and erythropoietic parameters are warranted to further clarify the complex relationship between maternal hematologic dynamics and NIPT performance.

## Data Availability

The original contributions presented in the study are included in the article/supplementary material, further inquiries can be directed to the corresponding author.
